# Improving diagnosis and broadening the phenotypes in early-onset seizure and severe developmental delay disorders through gene panel analysis

**DOI:** 10.1136/jmedgenet-2015-103263

**Published:** 2016-03-18

**Authors:** Natalie Trump, Amy McTague, Helen Brittain, Apostolos Papandreou, Esther Meyer, Adeline Ngoh, Rodger Palmer, Deborah Morrogh, Christopher Boustred, Jane A Hurst, Lucy Jenkins, Manju A Kurian, Richard H Scott

**Affiliations:** 1 North East Thames Regional Genetics Service, Great Ormond Street Hospital for Children, London, UK; 2 Molecular Neurosciences, Developmental Neurosciences Programme, University College London Institute of Child Health, London, UK; 3 Department of Neurology, Great Ormond Street Hospital for Children, London, UK; 4 Genetics and Genomic Medicine Unit, University College London Institute of Child Health, London, UK

**Keywords:** Epilepsy and seizures, Diagnostics, Copy-number

## Abstract

**Background:**

We sought to investigate the diagnostic yield and mutation spectrum in previously reported genes for early-onset epilepsy and disorders of severe developmental delay.

**Methods:**

In 400 patients with these disorders with no known underlying aetiology and no major structural brain anomaly, we analysed 46 genes using a combination of targeted sequencing on an Illumina MiSeq platform and targeted, exon-level microarray copy number analysis.

**Results:**

We identified causative mutations in 71/400 patients (18%). The diagnostic rate was highest among those with seizure onset within the first two months of life (39%), although overall it was similar in those with and without seizures. The most frequently mutated gene was *SCN2A* (11 patients, 3%). Other recurrently mutated genes included *CDKL5, KCNQ2*, *SCN8A* (six patients each), *FOXG1, MECP2, SCN1A, STXBP1* (five patients each), *KCNT1, PCDH19, TCF4* (three patients each) and *ATP1A3, PRRT2* and *SLC9A6* (two patients each). Mutations in *EHMT1, GABRB3, LGI1, MBD5, PIGA, UBE3A* and *ZEB2* were each found in single patients. We found mutations in a number of genes in patients where either the electroclinical features or dysmorphic phenotypes were atypical for the identified gene. In only 11 cases (15%) had the clinician sufficient certainty to specify the mutated gene as the likely cause before testing.

**Conclusions:**

Our data demonstrate the considerable utility of a gene panel approach in the diagnosis of patients with early-onset epilepsy and severe developmental delay disorders., They provide further insights into the phenotypic spectrum and genotype–phenotype correlations for a number of the causative genes and emphasise the value of exon-level copy number testing in their analysis.

## Introduction

Seizures affect approximately 7 in 10 000 children before the age of 2 years and 1 in 500 before 5 years and are often associated with developmental delay.^1–3^ An increasing number of causative genes are recognised in what is emerging as an overlapping group of disorders with varying prevalence and severity of seizures, developmental delay and, in some cases, dysmorphic features or congenital malformations.^4^ ^5^

Standard diagnostic approaches include biochemical and enzyme analysis for neurometabolic disorders, MRI brain imaging and genome-wide microarray analysis.^1^ Where these investigations do not identify a structural brain anomaly, biomarkers for a neurometabolic disorder, or chromosomal CNV, diagnosis is often challenging and has traditionally been dependent on the recognition of a characteristic phenotype followed by targeted single-gene testing. Examples include *SCN1A*-related seizure disorders and classical Rett syndrome (*MECP2*).^6^ ^7^

With increasing published literature on the wide spectrum of molecular aetiologies in these patients, targeted gene testing has allowed diagnosis in an increasing number of patients. Children with early-onset seizures and severe developmental delay (early infantile epileptic encephalopathy (EIEE)) can often be classified according to their electroclinical phenotype, for example, Ohtahara syndrome, West syndrome and epilepsy of infancy with migrating focal seizures (EIMFS).^8^ For some cases, accurate EIEE syndrome classification can assist in the identification of causative genes. Similarly, the recognition of a number of neurobehavioural and dysmorphic syndromes also helps identify disease-causing genes in some individuals.^9^ However, for many cases, clinical prediction of causative genes is challenging due to genetic heterogeneity, phenotypic pleiotropy, and for some patients, the phenotype is not sufficiently distinctive to accurately predict the causative gene. In such cases, sequential single-gene testing is time-consuming, costly and often unsuccessful.

With the increasing availability of next-generation sequencing technologies, it is becoming possible to rely less on targeted single-gene analysis. This has the potential to improve diagnostic rates. It can also clarify and broaden the phenotypic spectrum of genes by reducing ascertainment bias.^10^

We, therefore, chose to explore the diagnostic utility and genotype–phenotype insights provided by analysis of a panel of 46 genes in 400 patients with early-onset seizures and/or severe developmental delay.

## Methods

### Patients and samples

Four hundred patients with early-onset seizure disorders and/or severe developmental delay were referred and consented for gene panel testing by a paediatric neurologist or clinical geneticist, from a number of tertiary centres in the UK. Recruited patients did not have major structural brain malformations nor clinically significant copy number defects on microarray. Standardised clinical information was collected using a pre-test questionnaire completed by the recruiting clinician. Lymphocyte DNA was collected in all cases using standard procedures.

We selected genes for analysis in the panel that were established causes of early-onset seizures and/or severe developmental delay in patients without frequent major structural brain anomalies. Genes leading to neurometabolic disorders with readily identifiable blood/urine/cerebrospinal fluid (CSF) biomarkers were not included in the panel. The genes analysed were *ADSL, ALG13, ARHGEF9, ARX, ATP1A3, ATRX, CDKL5, CHD2, CHRNA4, CHRNB2, CNTNAP2, EHMT1, FOXG1, GABRB3, GRIN2A, GRIN2B, KCNQ2, KCNT1, KIAA1279, LGI1, MAGI2, MBD5, MECP2, MEF2C, NRXN1, PCDH19, PIGA, PLCB1, PNKP, POLG, PRRT2, SCN1A, SCN2A, SCN8A, SLC16A2, SLC25A22, SLC2A1, SLC9A6, SPTAN1, STXBP1, SYNGAP1, TBC1D24, TCF4, UBE2A, UBE3A* and *ZEB2*. We performed our analyses over 18 months in three phases, adding newly discovered genes to the panel as they were identified and employing improved sequence capture technology as it became available (see online supplementary table S1). In phase 1, 48 patients were analysed through 29 genes. In phase 2, 94 patients were analysed through 39 genes. In phase 3, 258 patients were analysed through 46 genes (see online supplementary table S1). Patients in phases 1 and 2 were not retested with later versions of the panel.

10.1136/jmedgenet-2015-103263.supp1 Supplementary tables

### Sequence analysis

All patients were sequenced using a MiSeq platform (Illumina). Parental DNA was only analysed to follow-up on potentially pathogenic variants (described below). Phase 1 cases were analysed using the Haloplex sequence capture system (Agilent). Phase 2 cases were analysed using the TrueSeq Custom Amplicon (TSCA) system (Illumina), and the phase 3 cases were analysed using the SureSelectXT system (Agilent). Sequence capture libraries were designed to capture the full transcript of the target genes plus 50 bps at each intron–exon boundary using the proprietary design tools, respectively. Libraries were captured using the standard protocols for each sequence capture system and sequencing performed using standard protocols for the MiSeq platform. Barcoding allowed multiplexing of 16 cases per flow cell.

We analysed sequence data using an in-house pipeline. Regions of interest were defined in BED file format by uploading human gene nomenclature committee (HGNC) genes names to the UCSC table browser.^11^ Sequence reads in FASTQ format were aligned to the reference human genome (hg19) using BWA (0.6.1-r104) and default settings.^12^ Variant calling was performed across the entire region of interest using VarScan2 (V.2.3.7) with the following settings: minimum 30× coverage, minimum five alternate reads, minimum phred-like base-quality of 20.^13^ ^14^ Variant calls in VCF format were then annotated using Ensembl Variant Effect Predictor (V.73) and the output parsed using an in-house script, which converts the annotated VCF file into Excel format for subsequent variant filtering and prioritisation.^15^ For each case, coverage was assessed across the coding exons of the target genes and their intron–exon boundaries (+6 bp and −12 bp) and expressed as the percentage of bases covered at ≥30×.

We used Sanger sequencing to analyse the commonly mutated GC-rich amplicon of the *ARX* gene that was not well targeted by our next-generation assays.

We also Sanger sequenced custom-designed amplicons to confirm potentially disease-causing variants in probands and perform segregation analysis in parental lymphocyte DNA samples. We performed PCR using MegaMix (Microzone, UK), purified PCR products using AmpureXP (Beckman Coulter, UK) and sequenced them using Big Dye Terminator V.3.1 Cycle Sequencing Kit (Applied Biosystems, USA). We used Mutation Surveyor (SoftGenetics, USA) to align traces to the reference sequence and call variants. Variant prioritisation and reporting was performed as a joint clinical laboratory process to optimise interpretation of both molecular and clinical data.

### Copy number analysis

In 33 mutation negative cases from phase 1, we performed exon-level copy number analysis using a custom Roche Nimblegen oligonucleotide 135K aCGH (see online supplementary table S1). Arrays were designed using proprietary Nimblegen software and read using a Roche Nimblegen MS 200 scanner. We analysed array data using CGH Fusion (infoQuant). We confirmed copy number abnormalities and performed segregation analysis in parental lymphocyte DNA samples using multiplex ligation-dependent probe amplification (MLPA) and/or FISH.

## Results

**Figure 1:**
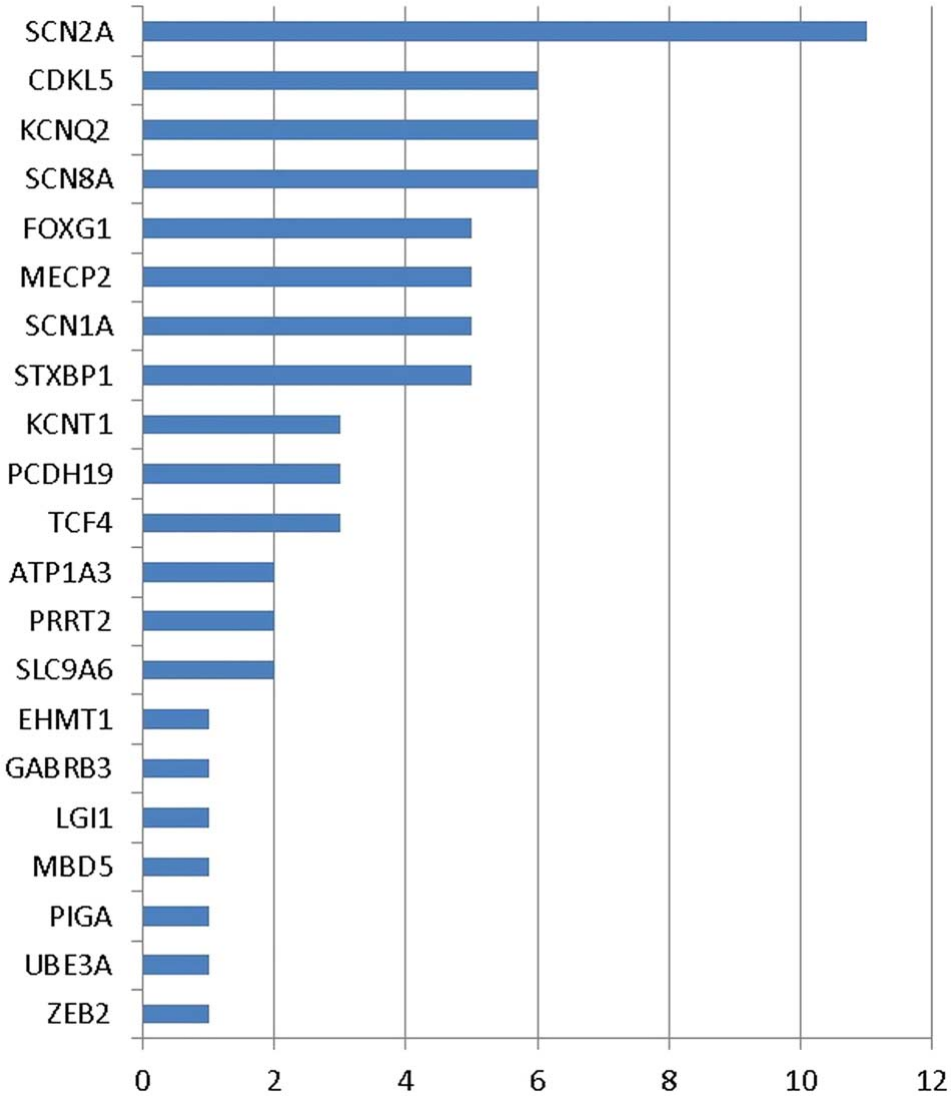
Number of cases with mutations in each gene.

We identified causative mutations in 71 patients (18%; see table 1, online supplementary table S1 and figure 1). The most frequently mutated gene was *SCN2A* (11 patients, 3%), occurring *de novo* in all patients. Other recurrently mutated genes included *CDKL5, KCNQ2*, *SCN8A* (six patients each), *FOXG1, MECP2, SCN1A, STXBP1* (five patients each), *KCNT1, PCDH19*, *TCF4* (three patients each) and *ATP1A3, PRRT2* and *SCL9A6* (two patients each). Mutations in *EHMT1, GABRB3, LGI1, MBD5, PIGA, UBE3A* and *ZEB2* were each found in single patients. Of the 54 patients for whom parental samples were available, 51 mutations were confirmed as *de novo.* For two patients, both harbouring *KCNQ2* variants, mutations were inherited from a similarly affected parent. One *FOXG1* variant had been inherited from the child's mother, who was mosaic for the mutation and clinically unaffected. Sixty-eight variants were either point mutations or small insertions and deletions. Three were larger *de novo* copy number abnormalities detected on our targeted array, a deletion encompassing *MBD5* and two other genes (confirmed by FISH using probe RP11-548K3), a single-exon deletion within *MECP2* and a multiexon duplication within *CDKL5* (both confirmed by MLPA using kits P015-E1 and P189-B1, respectively).

**Table 1:** Summary of the 400 patients analysed

	Cases	Cases with mutation	Percentage with mutation (%)
All cases	400	71	18
Male	192	41	21
Female	208	29	14
Seizures	323	60	19
Seizure onset <2 years	222	31	14
Seizure onset <2 months	77	30	39
Developmental delay, no seizures	77	11	14

Mutations were identified in patients with a broad range of phenotypes including EIEE syndromes, Dravet syndrome, neonatal seizures and a range of severe developmental delay phenotypes without seizures or with infrequent or resolved seizures (see tables 1 and 2 and online supplementary table S2). Mutations were identified in 60 of the 323 (18%) individuals with seizures, including 30 of the 76 (39%) with seizure onset under 2 months of age. Mutations were identified in 11 of the 77 (14%) without seizures.

**Table 2:** Summary of the 71 mutation-positive cases

Case	Gene	Mutation	Inheritance	Gender	Diagnosis at referral for testing	Seizures
1*	*ATP1A3*	het c.958G>A; p.(Ala320Thr)	*De novo*	M	Possible *ATP1A3*-related disorder	Yes
2	*ATP1A3*	het c.2839G>A; p.(Gly947Arg)	Not in mother	M	Developmental delay with seizures	Yes
3	*CDKL5*	hemi c.532C>T; p.(Arg178Trp)	*De novo*	M	EIEE	Yes
4	*CDKL5*	hemi c.2152G>A; p.(Val718Met)	Unknown	M	EIEE	Yes
5	*CDKL5*	hemi c.2177_2186delinsAATGTGTCAAC; p.(Ser726*)	*De novo*	M	EIEE	Yes
6	*CDKL5*	het duplication exons 6–11	*De novo*	F	EIEE	Yes
7	*CDKL5*	het c.167_168del; p.(Thr56Asnfs*6)	*De novo*	F	EIEE	Yes
8	*CDKL5*	het c.965del; p.(Thr322Asnfs*28)	Unknown	F	EIEE	Yes
9	*EHMT1*	het c.1596del; p.(Thr533Profs*30)	*De novo*	M	Developmental delay with seizures	Yes
10	*FOXG1*	het c.256dup; p.(Gln86Profs*35)	Unknown	M	EIEE	Yes
11	*FOXG1*	het c.572T>G; p.(Met191Arg)	From mosaic mother	M	Developmental delay with seizures	Yes
12	*FOXG1*	het c.651C>G; p.(Tyr217*)	*De novo*	F	Developmental delay	No
13	*FOXG1*	het c.695A>G ; p.(Asn232Ser)	*De novo*	M	Developmental delay with movement disorder	No
14	*FOXG1*	het c.1188C>A; p.(Cys396*)	*De novo*	M	Developmental delay with movement disorder	No
15	*GABRB3*	het c.860C>T; p.(Thr287Ile)	*De novo*	M	EIEE	Yes
16	*KCNQ2*	het c.601C>T; p.(Arg201Cys)	Unknown	F	EIEE	Yes
17	*KCNQ2*	het c.637C>T; p.(Arg213Trp)	Unknown	F	EIEE	Yes
18	*KCNQ2*	het c.638G>A; p.(Arg213Gln)	Unknown	M	EIEE	Yes
19	*KCNQ2*	het c.1681C>T; p.(Pro561Ser)	*De novo*	M	EIEE	Yes
20*	*KCNQ2*	het c.1741C>T; p.(Arg581*)	From father	M	Neonatal seizures	Yes
21*	*KCNQ2*	het c.1741C>T; p.(Arg581*)	From mother	M	Neonatal seizures	Yes
22	*KCNT1*	het c.862G>A; p.(Gly288Ser)	Unknown	M	Developmental delay with seizures	Yes
23	*KCNT1*	het c.2687T>A; p.(Met896Lys)	*De novo*	F	EIEE	Yes
24*	*KCNT1*	het c.2800G>A; p.(Ala934Thr)	*De novo*	M	EIMFS	Yes
25	*LGI1*	het c.1A>G p.(Met1?)	Unknown	M	EIEE	Yes
26	*MBD5*	het del chr2:149219863–149796844 including *MBD5*, *EPC2*, *KIF5C*	*De novo*	M	Developmental delay	Yes
27*	*MECP2*	het exon 4 deletion	*De novo*	F	Rett syndrome	Yes
28*	*MECP2*	het c.62+2_62+3del	Unknown	F	Possible Rett syndrome	No
29	*MECP2*	het c.844C>T; p.(Arg282*)	Unknown	F	Developmental delay with seizures	Yes
30*	*MECP2*	het c.952C>T; p.(Arg318Cys)	*De novo*	F	Rett syndrome	No
31	*MECP2*	het c.1119_1147del; p.(Lys375Leufs*20)	Unknown	F	Developmental delay with seizures	Yes
32	*PIGA*	het c.1064T>C; p.(Leu355Ser)	*De novo*	M	EIEE	Yes
33	*PCDH19*	het c.688G>A; p.(Asp230Asn)	*De novo*	F	EIEE	Yes
34	*PCDH19*	het c.707C>T; p.(Pro236Leu)	*De novo*	F	EIEE	Yes
35	*PCDH19*	het c.1882dup; p.(Arg628Profs*12)	*De novo*	F	EIEE	Yes
36*	*PRRT2*	het c.649dup; p.(Arg217Profs*8)	Unknown	M	Kinesogenic dyskinesia	No
37	*PRRT2*	het c.1021T>C; p.(*341Argext*28)	Unknown	F	Infantile seizure disorder	Yes
38	*SCN1A*	het c.302G>A; p.(Arg101Gln)	*De novo*	M	EIEE	Yes
39*	*SCN1A*	het c.2589+1_2589+2dup	*De novo*	M	Dravet syndrome	Yes
40	*SCN1A*	het c.3851G>A; p.(Trp1284*)	Unknown	M	Developmental delay with seizures	Yes
41	*SCN1A*	het c.4034C>T; p.(Pro1345Leu)	*De novo*	F	EIMFS	Yes
42	*SCN1A*	het c.5010_5013del; p.(Phe1671Thrfs*8)	*De novo*	F	EIEE	Yes
43	*SCN2A*	het c.2619C>G; p.(Ile873Met)	*De novo*	F	EIEE with movement disorder	Yes
44	*SCN2A*	het c.2960G>T; (p.Ser987Ile)	*De novo*	F	EIEE	Yes
45	*SCN2A*	het c.2995G>A; p.(Glu999Lys)	*De novo*	M	EIEE	Yes
46	*SCN2A*	het c.2996A>T; p.(Glu999Val)	*De novo*	F	EIEE	Yes
47	*SCN2A*	het c.3778A>G; p.(Lys1260Glu) and c.3778A>C; p.(Lys1260Gln) mosaic	*De novo*	M	EIEE	Yes
48	*SCN2A*	het c.4303C>T; p.(Arg1435*)	*De novo*	M	Autism with seizures	Yes
49	*SCN2A*	het c.4436A>C; p.(Gln1479Pro)	*De novo*	M	EIEE	Yes
50	*SCN2A*	het c.4949T>C; p.(Leu1650Pro)	*De novo*	M	EIEE	Yes
51	*SCN2A*	het c.5485C>T; p.(Leu1829Phe)	*De novo*	M	EIEE	Yes
52	*SCN2A*	het c.5645G>A; p.(Arg1882Gln)	*De novo*	M	EIEE	Yes
53	*SCN2A*	het c.5645G>A; p.(Arg1882Gln)	*De novo*	M	EIEE	Yes
54	*SCN8A*	het c.1222G>A; p.(Ala408Thr)	*De novo*	M	EIEE with movement disorder	Yes
55	*SCN8A*	het c.3943C>G; p.(Val1315Met)	*De novo*	F	EIEE	Yes
56	*SCN8A*	het c.3967G>T; p.(Ala1323Ser)	*De novo*	M	EIEE	Yes
57	*SCN8A*	het c.3979A>G; p.(Ile1327Val)	*De novo*	M	EIEE	Yes
58	*SCN8A*	het c.5261T>C; p.(Phe1754Ser)	**De novo**	M	EIEE	Yes
59	*SCN8A*	het c.5594T>C; p.(Leu1865Pro)	*De novo*	F	EIEE	Yes
60	*SLC9A6*	hemi c.608del p.(His203Leufs*10)	*De novo*	M	Developmental delay with seizures and movement disorder	Yes
61	*SLC9A6*	hemi c.1222_1226del; p.(His408Asnfs*2)	*De novo*	M	EIEE	Yes
62	*STXBP1*	het c.37+1G>A	*De novo*	F	EIEE	Yes
63	*STXBP1*	het c.842T>C; p.(Leu281Pro)	*De novo*	F	Developmental delay with seizures	Yes
64	*STXBP1*	het c.875G>A; p.(Arg292His)	*De novo*	M	EIEE	Yes
65	*STXBP1*	het c.1019_1020del; p.(Glu340Alafs*12)	Unknown	M	Neonatal seizures including infantile spasms	Yes
66	*STXBP1*	het c.1249+1G>T	Unknown	F	EIEE	Yes
67	*TCF4*	het c.826C>T; p.(Arg276*)	*De novo*	F	Developmental delay	No
68*	*TCF4*	het c.1065C>G; p.(Se355Arg)	*De novo*	F	Pitt–Hopkins syndrome	No
69	*TCF4*	het c.1296+1G>T	*De novo*	M	Developmental delay	No
70	*UBE3A*	het c.2572_2576dup; p.(Lys859Asnfs*7)	Unknown	F	Developmental delay	No
71*	*ZEB2*	het c.2083C>T; p.(Arg695*)	*De novo*	M	Mowat–Wilson syndrome	No

*Cases in which referring clinician correctly nominated the causative gene at referral for testing.

EIEE, early infantile epileptic encephalopathy; EIMFS, epilepsy of infancy with migrating focal seizures; F, female; M, male.

Mean coverage varied substantially between platforms, with a mean of 90.0% bases covered at ≥30× for samples analysed using the Haloplex system, 85.0% bases for the TSCA system and 99.8% for the SureSelect system.

## Discussion

Our panel detected clearly pathogenic mutations in 18% of our cases, demonstrating its significant diagnostic utility in patients with early-onset seizure disorders and/or severe developmental delay. This diagnostic rate is impressive given the large number of previous investigations that many of the cases had undergone. In many cases, this had included numerous sequential single-gene testing.

Mutations were found in patients with a broad range of phenotypes (tables 1 and 2). The mutation detection rate was similar in those with seizures (18%) and those without seizures (14%). The mutation detection rate was highest among those with early-onset seizures, before the age of 2 months (39%).

In only 11 cases (15%) had the clinician sufficient clinical certainty to specify on the pre-test questionnaire the actual mutated gene as the likely cause before genetic testing. These included one patient with an *ATP1A3* mutation, presenting with seizures, developmental delay and alternating hemiplegia (case 1); two with inherited *KCNQ2* mutations, both presenting with autosomal-dominant neonatal seizures (cases 20 and 21); one with a *KCNT1* mutation presenting with EIMFS (case 24); three with *MECP2* mutations presenting with classical Rett syndrome (cases 27, 28 and 30); one with a *PRRT2* mutation presenting with kinesogenic dyskinesia (case 36); one with an *SCN1A* mutation presenting with Dravet syndrome (case 39); one with a *TCF4* mutation presenting with dysmorphism characteristic of Pitt–Hopkins syndrome (case 68); and one with a *ZEB2* mutation presenting with facial dysmorphism and congenital malformations characteristic of Mowat–Wilson syndrome (case 71). However, it was clearly evident from analysis of the pre-test clinical proforma that even among these, the clinician often considered a wider range of genes as possibly causative for their patient's clinical phenotype.

In many of the remaining cases, panel analysis provided a diagnosis that would not otherwise have been reached using conventional approaches or reduced the time, number of investigations and cost to make the diagnosis. In turn, this allowed appropriate advice to be given on prognosis, the tailoring of medical management (eg, antiepileptic medication considered suitable for specific genotypes) and accurate advice on risks of recurrence for future pregnancies.

In a number of cases, electroclinical diagnoses considered strongly suggestive of the causative gene were only reached after re-examination of the phenotype in the light of the identified mutation. For example, we found a *KCNT1* mutation in a child later recognised to have classical EIMFS (case 23) rather than the initial diagnosis of epileptic encephalopathy with multifocal EEG discharges.

Similarly, in other cases, identification of the causative mutation allowed retrospective identification of a dysmorphic syndrome that is often considered recognisable. We identified *MECP2* mutations in two children whose clinical features were consistent with classical Rett syndrome but in whom it had not been considered the most likely causative gene (cases 29 and 31). We identified *TCF4* mutations in two children with severe developmental delay and facial features later confirmed to be in keeping with Pitt–Hopkins syndrome (cases 67 and 69). We also reported a *UBE3A* mutation in a child previously tested for Angelman methylation defects but in whom a *UBE3A* mutation was not considered sufficiently likely to warrant single-gene mutation analysis of *UBE3A* (case 70).

The majority of mutations were found in cases where the phenotype was either not easily distinguishable from that caused by a number of other genes or was atypical for the previously reported phenotype(s), emphasising the benefit of a gene panel approach over targeted single-gene testing. The extent to which more detailed pre-test phenotyping could ameliorate this varies between genes. Many of the patients described in this series had undergone extensive clinical and electroclinical workup by experts in these disorders prior to panel testing, illustrating the challenges in recognising the causative gene based on phenotype.

*De novo* monoallelic mutations in *SCN2A* are an increasingly recognised cause of an early-onset seizure and developmental delay.^16^ Despite its identification in 2001, testing of the gene has only become widely available with the advent of next-generation sequencing.^17^ *SCN2A* was the most frequently mutated gene in our series, accounting for 11 of 71 mutations and 3% of the series overall (figure 1 and table 2). The majority of mutations were *de novo* missense variants and occurred in cases with EIEE and severe or profound developmental delay. The median age of onset of seizures was 1 day of age (range 1 day to 2 years 11 months). The only child with a truncating mutation in *SCN2A* (case 48) presented with autism and clusters of seizures from 9 months of age. These observations provide further support for the proposed association of truncating mutations with a milder seizure phenotype.^16^ This child was one of two with only moderate developmental delay. A further child (case 47) was apparently mosaic for two different *de novo* missense variants at the same residue: c.3778A>G; p.(Lys1260Glu) and c.3778A>C; p.(Lys1260Gln). He had better seizure control than the other children with *SCN2A* missense mutations and a somewhat milder developmental phenotype, suggesting that one or both of the variants may be attenuating the phenotype. Case 43, a profoundly developmentally delayed 14-year-old girl with an *SCN2A* missense mutation, c.2619C>G; p.(Ile873Met), had developed a generalised dystonia/dyskinetic movement disorder and had an abnormal electroretinogram. These latter features have not previously been reported to our knowledge with *SCN2A* mutations. This child was also homozygous for the previously reported *PNKP* variant c.58C>T; p.(Pro20Ser).^18^ As discussed below, the contribution of this *PNKP* variant to her phenotype is uncertain.

*De novo* monoallelic missense mutations in *SCN8A* are a recently recognised cause of early-onset seizures with developmental delay.^19^ ^20^ We identified six such *SCN8A* mutations in our series. All patients had early onset seizures, with a median age of onset of 5 weeks of age (range 1 day to 5 months). In all cases, the electroclinical phenotype was relatively non-specific. In the five patients where we have data, the developmental profile is of severe or profound developmental delay. One child had a very good response to phenytoin. Other clinical and radiological manifestations may also be features of the condition. One child with profound developmental delay (case 54) had a spastic dystonic movement disorder aged 9 years. This child additionally had marked hypomyelination on MRI of the brain.

Monoallelic mutations in *KCNQ2* are a well-established cause of early-onset seizures. Reported phenotypes vary from benign familial neonatal seizures to a progressive pharmacoresistant EIEE.^21^ ^22^ We identified six *KCNQ2* mutations. All had had seizures from the neonatal period, with a median age of onset of 5 days. Two apparently unrelated individuals with benign neonatal seizures (cases 20 and 21) had the same truncating mutation, c.1741C>T p.(Arg581*), which had been inherited from a similarly affected parent.^23^ This mutation has been reported previously in a similar context. We identified four *de novo* missense mutations in children with EIEE, including two with mutations in the same codon, (c.637C>T; p.(Arg213Trp) and c.638G>A; p.(Arg213Gln). These findings add weight to previously reported genotype–phenotype observations for *KCNQ2*, with truncating mutations associated with the benign, inherited phenotype and missense mutations affecting key residues with the severe, sporadic phenotype. Cellular experiments indicate that these latter mutations may have a dominant negative effect on the function at a cellular level.^22^

*De novo* monoallelic mutations in *KCNT1* are known to cause early-onset seizures with developmental delay, with the typical presentation of EIMFS.^8^ ^24^ We identified three missense mutations in our series. While all presented with early-onset focal seizures, in only one child (case 24) had EIMFS been considered the working electroclinical diagnosis prior to genetic testing. In a second (case 23), re-evaluation of the phenotype in the light of the mutation confirmed a diagnosis of EIMFS.

*De novo* monoallelic mutations in *CDKL5* are a well-recognised cause of EIEE and severe, Rett-like developmental delay.^25^ ^26^ *CDKL5* was mutated in five patients in our series despite targeted diagnostic testing of the gene being available and frequently requested by referring clinicians. The gene is located on the X chromosome, and the majority of reports describe *de novo* X-linked dominant mutations in females.^25^ One mutation-positive girl in our series (case 6) had previously undergone diagnostic Sanger sequencing of the gene. This was not capable of detecting her *de novo* multiexon intragenic *CDKL5* duplication identified by our exon-level microarray. Of the remaining four *CDKL5* mutation-positive cases, three were male, highlighting the potential for under-recognition of this gene as a cause of disease in males. All male *CDKL5* mutation-positive cases had been classified as having an EIEE, presenting with seizures including infantile spasms aged 3 weeks and had severe or profound developmental delay. Two of these had confirmed *de novo* mutations, one a truncation and one missense mutation. The third (case 4), from whom parental samples are not available, had a mutation previously reported in an affected female.

*De novo* monoallelic mutations in *FOXG1* are typically associated with early-onset seizures and severe, Rett-like developmental delay.^27^ ^28^ Other typical features include agenesis or hypoplasia of the corpus callosum and a complex movement disorder with dyskinesia, dystonia and chorea. We found *FOXG1* mutations in five individuals. Only two had seizures and severe developmental delay (cases 10 and 12). The other three mutation-positive cases had a strikingly milder phenotype. Two manifested with moderate developmental delay with a movement disorder but no seizures (cases 13 and 14). A third (case 11) had mild developmental delay with (well-controlled) seizures from 4 years. His mother was somatic mosaic for the same missense mutation and was clinically unaffected. The mutation was also present in the proband's two similarly affected sisters. We postulate that the phenotype of *FOXG1* syndrome now includes a broader phenotypic spectrum including milder cases with prominent movement disorders. Our data may be in keeping with a genotype–phenotype correlation underlying this broadened phenotypic spectrum: of these milder cases, one had a nonsense mutation late in the gene (case 14). The other two had novel *de novo* missense variants (cases 11 and 13).

Hemizygous mutations in *SLC9A6* cause an X-linked recessive disorder known as Christianson syndrome.^29^ It was previously referred to as X-linked Angelman syndrome and presents with early-onset seizures, microcephaly, ataxia, dystonic movements and evolving lower limb spasticity from the second decade. The phenotype has been relatively consistent in the 15 families reported to date.^30^ We identified mutations in *SLC9A6* in two boys in our series. Both had typical features of the condition, with severe seizure disorders and severe or profound developmental delay and dystonic movements. However, the gene had not been considered the likely cause prior to testing in either boy. As such, our findings both support the existing description of the phenotype and emphasise the difficulty in recognising it. Although both of the mutations we identified were *de novo*, diagnosis of this X-linked recessive disorder can be associated with a high risk or recurrence in further sons of a carrier mother.

The large majority of mutations identified in our series were *de novo* (51 of 54 from whom parental samples were available). Partly, this reflects the fact that most of the genes analysed cause disease by *de novo* monoallelic mutations. This considerably aids analysis as rare variants of uncertain significance can usually be ruled out if they are inherited from an unaffected parent. Inherited variants pose greater challenges in interpretation, particularly in the absence of a characteristic phenotype or clear-cut prior evidence regarding the variant. Illustrative of these challenges are our findings in individuals with variants of uncertain significance in *LGI1* and *PNKP*.

Inherited monoallelic loss-of-function mutations in *LGI1* cause autosomal-dominant temporal lobe epilepsy.^31^ We identified an initiation codon mutation in *LGI1* in one child in our series who presented with EIEE including infantile spasms and subsequently frequent myoclonus and severe developmental delay. There was no specific evidence of temporal lobe seizures and no family history suggestive of temporal lobe epilepsy. His parents declined testing for the *LGI1* variant. While it is likely that the variant predisposes to temporal lobe epilepsy, we consider that it is unlikely to be the full explanation of his phenotype.

Biallelic loss-of-function mutations in *PNKP* cause an autosomal-recessive DNA repair disorder with a core phenotype of EIEE, developmental delay and microcephaly. Seventeen patients from nine families have been described to date.^18^ ^32^ ^33^ Six disease-causing variants have been reported, three frameshift variants and three missense variants. A number of these variants have been found in multiple families and/or have occurred in trans with other clearly pathogenic variants, providing further evidence of their pathogenicity. We identified one of the reportedly pathogenic missense variants, c.58C>T; p.(Pro20Ser), in 9 of our 400 cases. Its frequent occurrence in our cohort and our review of other available data lead us to be uncertain regarding the pathogenicity of this variant.

*PNKP* c.58C>T has been reported in one previous case with EIEE without data regarding head circumference.^18^ We identified the variant in homozygous form in one child. This child, case 43, also has a *de novo* monoallelic *SCN2A* mutation. She had EIEE, profound developmental delay and microcephaly, features that could be accounted for alone by the *SCN2A* mutation. In eight others, from a range of ethnic backgrounds, we identified the variant in heterozygous form without a second mutation despite good quality sequence data across the gene. One of these (case 40) also had an *SCN1A* nonsense mutation, consistent with the presenting phenotype. We note that the variant has an allele frequency of 0.0091 on the Exome Variant Server (http://evs.gs.washington.edu/EVS/) and has an allele frequency of 0.0076 on the ExAC database (http://www.exac.broadinstitute.org). These figures are similar to that in our series (0.0125). Taken together, these data suggest caution in interpreting this variant as disease causing. It is likely that this is a benign polymorphism.

The cost of gene panel analysis in a diagnostic setting is now similar to that of Sanger sequencing a single gene. Therefore, while targeted single-gene Sanger sequencing may remain appropriate in some cases, for example, typical *SCN1A*-related phenotypes, EIMFS (*KCNT1*), classical Rett syndrome (*MECP2*) and some cases with other dysmorphic syndromes, our data argue for the use of panel-based analysis as the diagnostic genetic test of choice in the majority of individuals with early-onset seizure and severe developmental delay disorders.

With time, we expect that panel analysis will evolve to use data generated from sequencing larger numbers of genes, for example, the whole exome or the clinical exome (all known disease genes).^10^ ^34^ Potential advantages include the ability to add newly discovered genes without the need to redesign the assay and to analyse larger panels of genes without running a separate test. However, we currently find the better sequence coverage offered by a carefully designed and curated panel outweighs these potential benefits in a diagnostic setting. We note that the mutation detection rate we report in our early-onset seizure cases is similar to that reported in studies employing whole-exome sequencing in similar cohorts.^35^

While next-generation sequencing is a relatively new technology, in many respects it is equally as robust as Sanger sequencing. In some respects, it may be more robust, for example, in the detection of low-level mosaic mutations.^34^ We identified two mutations that had been missed despite previous Sanger sequencing of the gene in question. An *SCN1A* mutation (case 39) had been missed due to a technical interpretation error, less likely with more bioinformatics-driven interpretation pipelines used in the analysis of next-generation sequencing data. A *MECP2* mutation (c.62+2_62+3delTG, in case 28) had also been missed because it affected the first exon of the gene, which many diagnostic laboratories have not sequenced until recently due to uncertainty about the most clinically relevant transcript.^36^

Like Sanger sequencing, next-generation sequencing remains poor at detecting exon-level copy number abnormalities, particularly when samples are run in the relatively small batches typical of a diagnostic setting.^34^ We, therefore, piloted the use of an exon-level microarray targeting our gene panel in a subset of our series. In our analysis of 33 cases without mutations on panel sequencing, we identified three likely pathogenic copy number abnormalities. Exon-level copy number analysis is also valuable in individuals harbouring single mutations in autosomal-recessive genes to search for second mutations and assist in the interpretation of the significance of sequence variants. Until next-generation sequencing allows robust copy number analysis, exon-level microarray will be a useful component of gene panel analysis and we have now integrated it into our standard analysis work stream.

In conclusion, our work demonstrates the significant utility of a gene panel approach in the diagnosis of patients with early-onset epilepsy and severe developmental delay disorders, particularly the former. It also broadens the phenotypic spectrum, provides insights into the genotype–phenotype relationships of a number of the causative genes and emphasises the importance of exon-level copy number testing in their analysis.
