# Supplementary material for: Improving diagnosis and broadening the phenotypes in early-onset seizure and severe developmental delay disorders through gene panel analysis
Source: J Med Genet. 2016 Mar 18;53(5):310–7. doi: 10.1136/jmedgenet-2015-103263 (PMC4862068; doi:10.1136/jmedgenet-2015-103263)
Supplement: Supplementary tables [file jmedgenet-2015-103263supp_tables.pdf]

Supplementary Table 1 Details of genes analysed in phases 1-3 of the study

Gene	Transcript	OMIM gene ID	Disorder	Inheritance	Phase 1 (Haloplex)	Phase 2 (TSCA)	Phase 3 (SureSelect)
ADSL	NM_000026	608222	Adenylosuccinase deficiency	AR	Yes	Yes	Yes
ARHGEF9	NM_015185	300429	Early infantile epileptic encephalopathy 8	XR	Yes	Yes	Yes
ARX	NM_139058	300382	Early infantile epileptic encephalopathy 1 and others	XR	Yes	Yes	Yes
ATRX	NM_000489	300032	Alpha-thalassaemia/mental retardation syndrome, X-linked	XR	Yes	Yes	Yes
CDKL5	NM_003159	300203	Early infantile epileptic encephalopathy 2	X	Yes	Yes	Yes
CNTNAP2	NM_014141	604569	Pitt-Hopkins-like syndrome 1	AR	Yes	Yes	Yes
EHMT1	NM_024757	607001	Kleefstra syndrome	AD	Yes	Yes	Yes
FOXG1	NM_005249	164874	Rett syndrome, congenital variant	AD	Yes	Yes	Yes
KCNQ2	NM_172107	602235	Early infantile epileptic encephalopathy 7 and Benign familial neonatal seizures	AD	Yes	Yes	Yes
KIAA1279	NM_015634	609367	Goldberg-Shprintzen megacolon syndrome	AR	Yes	Yes	Yes
MAGI2	NM_012301	606382	Infantile spasms	AD	Yes	Yes	Yes
MBD5	NM_018328	611472	2q23.1 microdeletion syndrome	AD	Yes	Yes	Yes
MECP2	NM_001110792	300005	Rett syndrome	X	Yes	Yes	Yes
MEF2C	NM_002397	600662	Mental retardation, stereotypic movements, epilepsy and/or cerebral malformations	AD	Yes	Yes	Yes
NRXN1	NM_001135659	600565	Pitt-Hopkins-like syndrome 2	AR	Yes	Yes	Yes
PCDH19	NM_001184880	300460	Early infantile epileptic encephalopathy 9	XD	Yes	Yes	Yes
PLCB1	NM_015192	607120	Early infantile epileptic encephalopathy 12	AR	Yes	Yes	Yes

Gene	Transcript	OMIM gene ID	Disorder	Inheritance	Phase 1 (Haloplex)	Phase 2 (TSCA)	Phase 3 (SureSelect)
PNKP	NM_007254	605610	Early infantile epileptic encephalopathy 10	AR	Yes	Yes	Yes
SCN1A	NM_001165963	182389	Dravet syndrome and others	AD	Yes	Yes	Yes
SCN2A	NM_021007	182390	Early infantile epileptic encephalopathy 11	AD	Yes	Yes	Yes
SLC16A2	NM_006517	300095	Allan-Herndon-Dudley syndrome	XR	Yes	Yes	Yes
SLC25A22	NM_001191060	609302	Early infantile epileptic encephalopathy 3	AR	Yes	Yes	Yes
SLC2A1	NM_006516	138140	GLUT1 deficiency syndrome	AR/AD	Yes	Yes	Yes
SLC9A6	NM_001042537	300231	Angelman-like syndrome, X-linked	XR	Yes	Yes	Yes
SPTAN1	NM_001130438	182810	Early infantile epileptic encephalopathy 5	AD	Yes	Yes	Yes
STXBP1	NM_003165	602926	Early infantile epileptic encephalopathy 4	AD	Yes	Yes	Yes
TCF4	NM_001083962	602272	Pitt-Hopkins syndrome	AD	Yes	Yes	Yes
UBE3A	NM_000462	601623	Angelman syndrome	Imprinted	Yes	Yes	Yes
ZEB2	NM_014795	605802	Mowat-Wilson syndrome	AD	Yes	Yes	Yes
ATP1A3	NM_152296	182350	Alternating hemiplegia of childhood 2 and others	AD		Yes	Yes
CHRNA4	NM_000744	118504	Epilepsy, nocturnal frontal lobe, 1	AD		Yes	Yes
CHRNB2	NM_000748	118507	Epilepsy, nocturnal frontal lobe, 3	AD		Yes	Yes
KCNT1	NM_020822	608167	Early infantile epileptic encephalopathy 14	AD		Yes	Yes
LGI1	NM_005097	604619	Epilepsy, familial temporal lobe, 1	AD		Yes	Yes
POLG	NM_002693	174763	Mitochondrial DNA depletion syndrome	AR		Yes	Yes
PRRT2	NM_145239	614386	Familial infantile convulsions with paroxysmal choreoathetosis	AD		Yes	Yes
SCN8A	NM_014191	600702	Early infantile epileptic encephalopathy 13	AD		Yes	Yes
SYNGAP1	NM_006772	603384	Mental retardation, autosomal dominant 5	AD		Yes	Yes

Gene	Transcript	OMIM gene ID	Disorder	Inheritance	Phase 1 (Haloplex)	Phase 2 (TSCA)	Phase 3 (SureSelect)
UBE2A	NM_003336	312180	Mental retardation, X-linked syndromic, Nascimento-type	XR		Yes	Yes
ALG13	NM_001099922	300776	Congenital disorder of glycosylation, type Is	AD			Yes
CHD2	NM_001271	602119	Epileptic encephalopathy, childhood-onset	AD			Yes
GABRB3	NM_000814	137192	Epilepsy, childhood absence, susceptibility to, 5	AD			Yes
GRIN2A	NM_001134407	138253	Epilepsy, focal, with speech disorder and with or without mental retardation	AD			Yes
GRIN2B	NM_000834	138252	Mental retardation, autosomal dominant 6	AD			Yes
PIGA	NM_002641.3	311770	Multiple congenital anomalies-hypotonia-seizures syndrome 2	XR			Yes
TBC1D24	NM_001199107	613577	Early infantile epileptic encephalopathy 16	AR			Yes

AD, autosomal dominant; AR, autosomal recessive; XR, X-linked recessive; X, X-linked dominant or recessive; TSCA, TrueSeq Custom Amplicon

Supplementary Table 2 Detailed clinical information on mutation positive cases

**Case**	**Gene**	**Mutation**	**Inheritance**	**Gender**	**Diagnosis at referral**	**Seizures**	**Age of onset**	**Seizure types / EEG findings**	**Developmental delay**	**Dysmorphic**	**Microcephaly (<2nd centile)**	**Other medical complications or findings on investigation**	**Comment**
1	*ATP1A3*	het c.958G>A; p.(Ala320Thr)	*De novo*	M	Possible *ATP1A3*-related disorder	Yes	6m	Tonic-clonic seizures	Profound	No	No	Paroxysmal movement disorder; neonatal distal athrogyposis	
2	*ATP1A3*	het c.2839G>A; p.(Gly947Arg)	Not in mother	M	Developmental delay with seizures	Yes	5m	Generalised with eye deviation	Mild	No	No	Alternating hemiplegia; dystonia	
3	*CDKL5*	hemi c.532C>T; p.(Arg178Trp)	*De novo*	M	EIEE	Yes	3w	Infantile spasms; subsequently myolclonic jerks	Profound	No	Yes		
4	*CDKL5*	hemi c.2152G>A; p.(Val718Met)	Unknown	M	EIEE	Yes	3w	Infantile spasms; subsequently tonic-clonic seizures; EEG initially hypsarrhthmia; subsequently multi-focal discharges	Severe	No data	No data		
5	*CDKL5*	hemi c.2177_2186delinsAATGTGTCAAC;	*De novo*	M	EIEE	Yes	No data	Infantile spasms;	Severe	No	No		

		p.(Ser726*)						subsequently atypical absences; myoclonic jerks					
6	*CDKL5*	het duplication exons 6-11	*De novo*	F	EIEE	Yes	1.25	Tonic; spasms; head drops; generalised abnormalities on EEG	Severe	No	No		
7	*CDKL5*	het c.167_168del; p.(Thr56Asnfs*6)	*De novo*	F	EIEE	Yes	3w	Infantile spasms; subsequently complex myoclonic jerks, absences; hypsarrhythmia	Severe	No	No	MRI brain: increased signal basal ganglia	
8	*CDKL5*	het c.965del; p.(Thr322Asnfs*28)	Unknown	F	EIEE	Yes	6w	Tonic; spasms; drug responsive	Severe	No	No		
9	*EHMT1*	het c.1596del; p.(Thr533Profs*30)	*De novo*	M	Developmental delay with seizures	Yes	5y	Staring episodes	Severe	Synophrys; prognathism	Yes		
10	*FOXG1*	het c.256dup; p.(Gln86Profs*35)	Unknown	M	EIEE	Yes	5w	No data	Severe	No	No		
11	*FOXG1*	het c.572T>G; p.(Met191Arg)	From mosaic mother	M	Developmental delay with seizures	Yes	4y	No data	Mild	No	No		
12	*FOXG1*	het c.651C>G; p.(Tyr217*)	*De novo*	F	Developmental delay	No	N/A	N/A	Severe	No	Yes		
13	*FOXG1*	het c.695A>G ; p.(Asn232Ser)	*De novo*	M	Developmental delay with movement	No	N/A	N/A	Moderate	No	No	Dystonic movements; myoclonus; tics	

					disorder								
14	*FOXG1*	het c.1188C>A; p.(Cys396*)	*De novo*	M	Developmental delay with movement disorder	No	N/A	N/A	Moderate	No	Yes	Dystonic movements; MRI brain: hypoplastic corpus callosum and absent septum pellucidum	
15	*GABRB3*	het c.860C>T; p.(Thr287Ile)	*De novo*	M	EIEE	Yes	3m	Staring episodes; tonic	Severe	No	No		
16	*KCNQ2*	het c.601C>T; p.(Arg201Cys)	Unknown	F	EIEE	Yes	<28d	Neonatal seizures; persistent generalised tonic spasms; burst suppression on early EEGs	Profound	No	Yes		
17	*KCNQ2*	het c.637C>T; p.(Arg213Trp)	Unknown	F	EIEE	Yes	2d	Neonatal seizures; burst suppression	No data	No	No		
18	*KCNQ2*	het c.638G>A; p.(Arg213Gln)	*De novo*	M	EIEE	Yes	1d	Neonatal seizures; tonic/tonic-clonic; focal abnormalities, particularly posteriorly	No data	No	No	MRI brain: thin corpus callosum	
19	*KCNQ2*	het c.1681C>T; p.(Pro561Ser)	*De novo*	M	EIEE	Yes	1d	Neonatal seizures; initially	Severe	No	No	Bilateral undescended	

								tonic; subsequently tonic-clonic; burst suppression on early EEG				testes	
20	*KCNQ2*	het c.1741C>T; p.(Arg581*)	Inherited from father	M	Neonatal seizures	Yes	8d	No data	No data	No data	No data		Family history of neonatal seizures
21	*KCNQ2*	het c.1741C>T; p.(Arg581*)	Inherited from mother	M	Neonatal seizures	Yes	8d	Neonatal seizures; initially clonic; subsequently focal motor seizures with secondary generalisation	Not delayed	No	No		Family history neonatal seizures including in mother, maternal aunt and grandfather
22	*KCNT1*	het c.862G>A; p.(Gly288Ser)	Unknown	M	Developmental delay with seizures	Yes	3m	Focal seizures with secondary generalisation	Severe	Flat midface, thin upper lip	No		
23	*KCNT1*	het c.2687T>A; p.(Met896Lys)	*De novo*	F	EIEE	Yes	5w	MPSI	Severe	No	No		
24	*KCNT1*	het c.2800G>A; p.(Ala934Thr)	*De novo*	M	MPSI	Yes	3w	MPSI	Severe	No	No		
25	*LGI1*	het c.1A>G p.(Met1?)	Unknown	M	EIEE	Yes	5m	Infantile spasms; myoclonic jerks	Severe	Deep-set eyes; upturned earlobes; hypoplastic alae nasi	Yes	Diabetes insipidus; cataracts	Likely incidental finding; no FHx epilepsy
26	*MBD5*	het del *MBD5, EPC2, KIF5C* del by	*De novo*	M	Developmental	Yes	2y	Single febrile	Severe	Flat midface,	Yes		

		array ; chr2:149,219,863-149,796,844			delay			seizure aged 2y		tented upper lip, broad hands, short distal phalanges			
27	*MECP2*	het exon 4 deletion	*De novo*	F	Rett syndrome	Yes	6y	Tonic-clonic seizures	Severe	No	No	Cyclical vomiting	
28	*MECP2*	het c.62+2_62+3del	Unknown	F	Developmental delay	No	N/A	N/A	Severe	No	Yes		*MECP2* mutation missed by Sanger sequencing
29	*MECP2*	het c.844C>T; p.(Arg282*)	Unknown	F	Devevelopmental delay with seizures	Yes	2y	Tonic-clonic seizures	Severe	No	Yes		
30	*MECP2*	het c.952C>T; p.(Arg318Cys)	*De novo*	F	Rett syndrome	No	N/A	N/A	Severe	Broad nasal bridge; thin upper lip	Yes		
31	*MECP2*	het c.1119_1147del; p.(Lys375Leufs*20)	Unknown	F	Developmental delay with seizures	Yes	9y	Complex partial, generalised tonic-clonic, astatic, myoclonic and tonic seizures	Severe	No	No	MRI brain: progressive cerebellar atrophy	
32	*PIGA*	het c.1064T>C; p.(Leu355Ser)	*De novo*	M	EIEE	Yes	5m	Myoclonic seizures	Severe	No	Yes	MRI brain: thin corpus callosum, reduced white matter bulk, signal change in dorsal pons, early	

												hippocampal sclerosis	
33	*PCDH19*	het c.688G>A; p.(Asp230Asn)	*De novo*	F	EIEE	Yes	10m	Clusters of tonic seizures	Severe	No data	No data	Developmental regression associated with clusters of seizures	
34	*PCDH19*	het c.707C>T; p.(Pro236Leu)	*De novo*	F	EIEE	Yes	8m	Focal seizures; multi-focal EEG abnormalities	Mild	No data	No data		
35	*PCDH19*	het c.1882dup; p.(Arg628Profs*12)	*De novo*	F	EIEE	Yes	11m	Mulitfocal seizures	Severe	No data	No data		
36	*PRRT2*	het c.649dup; p.(Arg217Profs*8)	Unknown	M	Kinesogenic dyskinesia	No	N/A	N/A	Mild	No	No		
37	*PRRT2*	het c.1021T>C; p.(*341Argext*28)	Unknown	F	Infantile seizure disorder	Yes	3m	Focal seizures	Not delayed development	No	No		
38	*SCN1A*	het c.302G>A; p.(Arg101Gln)	*De novo*	M	EIEE	Yes	1d	Focal, myoclonic and absence seizures; encephalopathic EEG	Severe	Hypertelorism, flat midface, bilateral single palmar creases	Yes		
39	*SCN1A*	het c.2589+1_2589+2dup	*De novo*	M	Dravet syndrome	Yes	6m	Generalised tonic-clonic, focal, absence seizures and non-convulsive status epilepticus	Moderate	No	No data		*SCN1A* mutation missed by Sanger sequencing
40	*SCN1A*	het c.3851G>A; p.(Trp1284*)	Unknown	M	Developmental delay with	Yes	1y	Generalised tonic-clonic	Severe	Flat midface, high nasal	No		Family history of

					seizures			seizures		bridge, narrow palpebral fissures			seizures; PNKP het c.58C>T; p.(Pro20Ser)
41	*SCN1A*	c.4034C>T; p.(Pro1345Leu)	*De novo*	F	MPSI	Yes	1d	Migrating partial seizures	Severe	No	No	Arthrogryposis	
42	*SCN1A*	het c.5010_5013del; p.(Phe1671Thrfs*8)	*De novo*	F	EIEE	Yes	6m	Generalised tonic-clonic, focal and atypical absence seizures	Mild	No	No		
43	*SCN2A*	het c.2619C>G; p.(Ile873Met)	*De novo*	F	EIEE with movement disorder	Yes	<28d	Neonatal seizures; subsequently tonic, absence and possibly gelastic	Profound	No	Yes	Generalised dystonia and dyskinesias; MRI brain: lack of white matter bulk; small hippocampi. Abnormal electroretinogram	PNKP hom c.58C>T; p.(Pro20Ser)
44	*SCN2A*	het c.2960G>T; p.(Ser987Ile)	*De novo*	F	EIEE	Yes	1d	Neonatal seizures with burst suppression and multifocal spiking on EEG; subsequently, focal seizures with secondary generalisation;	Severe	No	No		

								seizure free since 1y					
45	*SCN2A*	het c.2995G>A; p.(Glu999Lys)	*De novo*	M	EIEE	Yes	1m	MPSI	Severe	No	No		
46	*SCN2A*	het c.2996A>T; p.(Glu999Val)	*De novo*	F	EIEE	Yes	1d	Neonatal seizures	Severe	No	Yes		
47	*SCN2A*	het c.3778A>G; p.(Lys1260Glu) and c.3778A>C; p.(Lys1260Gln) mosaic	*De novo*	M	EIEE	Yes	3d	Neonatal focal seizures; subsequently multifocal; focal changes on EEG; seizure free since 1y	Moderate	No	No		
48	*SCN2A*	het c.4303C>T; p.(Arg1435*)	*De novo*	M	Autism with seizures	Yes	2y11m	Clusters of tonic-clonic seizures	Moderate	No	No	Autism spectrum disorder diagnosed prior to seizures	
49	*SCN2A*	het c.4436A>C; p.(Gln1479Pro)	*De novo*	M	EIEE	Yes	1d	Neonatal seizures; subsequently tonic, startle seizures	Profound	No	No	MRI brain: progressive cerebral and cerebellar atrophy; bilateral symmetrical signal abnormality within the ventral lateral thalami and also within the corticospinal tracts	
50	*SCN2A*	het c.4949T>C; p.(Leu1650Pro)	*De novo*	M	EIEE	Yes	9m	Generalised	Severe	No	No	Bilateral cataracts	

								myoclonic, tonic and absence seizures					
51	*SCN2A*	het c.5485C>T; p.(Leu1829Phe)	*De novo*	M	EIEE	Yes	1d	Neonatal seizures; subsequently tonic-clonic seizures	Severe	No	Yes		
52	*SCN2A*	het c.5645G>A; p.(Arg1882Gln)	*De novo*	M	EIEE	Yes	1d	Neonatal focal seizures; subsequently spasms, tonic, tonic-clonic, gelastic seizures	Severe	No	Yes		
53	*SCN2A*	het c.5645G>A; p.(Arg1882Gln)	*De novo*	M	EIEE	Yes	1d	Neonatal seizures; subsequently tonic, apnoeic seizures; burst suppression EEG	Severe	No	No		
54	*SCN8A*	het c.1222G>A; p.(Ala408Thr)	*De novo*	M	EIEE with movement disorder	Yes	1d	Neonatal seizures; subsequently tonic, clonic, possibly gelastic seizures	Profound	Yes	Yes	Spastic dystonic movement disorder; MRI brain: hypomyelination	
55	*SCN8A*	het c.3943C>G; p.(Val1315Met)	*De novo*	F	EIEE	Yes	4m	Tonic-clonic and tonic seizures	Severe	No	Yes		
56	*SCN8A*	het c.3967G>T; p.(Ala1323Ser)	*De novo*	M	EIEE	Yes	9w	Tonic and clonic seizures, often	Severe	No	No data		

								in clusters; EEG encephalopathic during seizure cluster					
57	*SCN8A*	het c.3979A>G; p.(Ile1327Val)	*De novo*	M	EIEE	Yes	1d	Neonatal seizures	No data	No	Yes		
58	*SCN8A*	het c.5261T>C; p.(Phe1754Ser)	*De novo*	M	EIEE	Yes	1d	Neonatal seizures; subsequently tonic seizures; EEG non-specific	Severe	No	No		
59	*SCN8A*	het c.5594T>C; p.(Leu1865Pro)	*De novo*	F	EIEE	Yes	4m	Tonic seizures in clusters	Severe	No	No		
60	*SLC9A6*	hemi c.608del p.(His203Leufs*10)	*De novo*	M	Developmental delay with seizures and movement disorder	Yes	12m	Clusters of tonic-clonic seizures; possibly gelastic	Severe	No	Yes	Dystonic movements	
61	*SLC9A6*	hemi c.1222_1226del; p.(His408Asnfs*2)	*De novo*	M	EIEE	Yes	8m	Generalised tonic-clonic, tonic and atypical absence seizures	Profound	No	Yes	Dystonic movements	
62	*STXBP1*	het c.37+1G>A	*De novo*	F	EIEE	Yes	3d	Neonatal seizures; burst suppression on early EEGs	N/A	No	No		
63	*STXBP1*	het c.842T>C; p.(Leu281Pro)	*De novo*	F	Developmental delay with seizures	Yes	no data	Multiple seizure types	Severe	Angelman-like facial appearance	No data		

64	*STXBP1*	het c.875G>A; p.(Arg292His)	*De novo*	M	EIEE	Yes	no data	Burst suppression EEG	Severe	No data	No data		
65	*STXBP1*	het c.1019_1020del; p.(Glu340Alafs*12)	Unknown	M	Neonatal seizures including infantile spasms	Yes	4d	Neonatal seizures evolving to infantile spasms; hypsarrhythmia on EEG during spasms	Moderate	No	No		
66	*STXBP1*	het c.1249+1G>T	Unknown	F	EIEE	Yes	4d	Neonatal seizures evolving to infantile spasms; non-specific EEG	Severe	No	No		
67	*TCF4*	het c.826C>T; p.(Arg276*)	*De novo*	F	Developmental delay	No	N/A	N/A		No	No		
68	*TCF4*	het c.1065C>G; p.(Se355Arg)	*De novo*	F	Pitt-Hopkins syndrome	No	N/A	N/A	Severe	Coarse facial features; everted lips	Yes		
69	*TCF4*	het c.1296+1G>T	*De novo*	M	Developmental delay	No	N/A	N/A	Severe	Wide mouth; everted lips with narrow lateral vermillion	Yes		
70	*UBE3A*	het c.2572_2576dup; p.(Lys859Asnfs*7)	Unknown	F	Developmental delay	No	N/A	EEG not typical for Angelman syndrome	Severe	Wide mouth	Yes		
71	*ZEB2*	het c.2083C>T; p.(Arg695*)	*De novo*	M	Mowat-Wilson	No	N/A	N/A	Severe	Upturned	Yes	Hirschsprung	

					syndrome					earlobes, prominent chin, deep-set eyes		disease; hypospadias	

M, male; F, female; EIEE, early infantile epileptic encephalopathy; MPSI, migrating partial seizures of infancy; d, day; m, month; y, yearl N/A, not available.
